# Analysis of the transcriptional regulation of cancer-related genes by aberrant DNA methylation of the cis-regulation sites in the promoter region during hepatocyte carcinogenesis caused by arsenic

**DOI:** 10.18632/oncotarget.4085

**Published:** 2015-05-25

**Authors:** Zhuang Miao, Lin Wu, Ming Lu, Xianzhi Meng, Bo Gao, Xin Qiao, Weihui Zhang, Dongbo Xue

**Affiliations:** ^1^ Department of General Surgery, The First Affiliated Hospital of Harbin Medical University, Harbin, PR China; ^2^ Department of Surgery, David Geffen School of Medicine, University of California at Los Angeles, Los Angeles, CA, USA

**Keywords:** carcinogenesis, DNA methylation, arsenic exposure

## Abstract

Liver is the major organ for arsenic methylation metabolism and may be the potential target of arsenic-induced cancer. In this study, normal human liver cell was treated with arsenic trioxide, and detected using DNA methylation microarray. Some oncogenes, tumor suppressor genes, transcription factors (TF), and tumor-associated genes (TAG) that have aberrant DNA methylation have been identified. However, simple functional studies of genes adjacent to aberrant methylation sites cannot well reflect the regulatory relationship between DNA methylation and gene transcription during the pathogenesis of arsenic-induced liver cancer, whereas a further analysis of the cis-regulatory elements and their trans-acting factors adjacent to DNA methylation can more precisely reflect the relationship between them. *MYC* and *MAX* (*MYC* associated factor X) were found to participating cell cycle through a bioinformatics analysis. Additionally, it was found that the hypomethylation of cis-regulatory sites in the *MYC* promoter region and the hypermethylation of cis-regulatory sites in the *MAX* promoter region result in the up-regulation of *MYC* mRNA expression and the down-regulation of *MAX* mRNA, which increased the hepatocyte carcinogenesis tendency.

## INTRODUCTION

As a cytotoxic substance, arsenic is widely existed in natural environment, particularly in Asian. Long-term exposure to a certain level of arsenic that can result in poisoning has been established as an issue that seriously harms individuals’ health [1]. Arsenic not only causes nerve and skin lesions but is also centrally involved in liver damage, cirrhosis and the pathogenesis of liver cancer [2]. Studies have shown that the liver is an important target organ of the carcinogenic effects of arsenic. A study by Li J [3] proposed that the liver was the potential target of arsenic-induced cancer, Lin HJ [4] reported that chronic arsenic poisoning through drinking water could promote the pathogenesis of liver cancer, Chen H [5] reported that long-term exposure to arsenic could induce abnormal gene expression and develop liver cancer. Previous studies have explored the mechanism of arsenic-induced hepatocarcinogenesis. For example, studies have confirmed that arsenic can directly or indirectly injure hepatocytes through DNA damage, lipid peroxidation, the inhibition of apoptosis and by promoting excessive cell proliferation [6]. Recent reports found that during its hepatocarcinogenesis process, arsenic stimulated the hypomethylation (HypoM) of *DNMTS* and *HA-RAS* genes [7] and the hypermethylation (HyperM) of *P16* and *P53* genes [3], indicating that the effect of arsenic on hepatocyte gene methylation levels may be an important mechanism during its hepatocarcinogenesis process.

Inorganic arsenic toxicity is decreased inside the body through methylation, and the liver is the major location for methylation metabolism. The methylation metabolism process of inorganic arsenic in the body competes for methyl donors with the DNA methylation modification process, which affects the DNA methylation-demethylation modification. With the increase of the accumulation and uptake of inorganic arsenic, the human body, particularly the liver, has a larger burden for arsenic methylation. Therefore, we speculate that under the conditions of long-term arsenic exposure, the methylation level of hepatocytes may change, which in turn affects the expression of proto-oncogenes and tumor suppressor genes, thereby increasing the tendency of hepatocarcinogenesis. To verify this inference, we performed DNA methylation detection and analysis using a methylation microarray on normal human liver cells that have been long-term exposed to arsenic to explore the possible mechanism for the pathogenesis of arsenic-induced liver cancer.

## RESULTS

### Analysis of the aberrant DNA methylation sites and adjacent gene annotation

Based on the analysis results of the aberrant DNA methylation sites, we detected that the DNA methylation signal in the arsenic-exposure group was up-regulated in 1148 DNA methylation sites, which represents the HyperM phenomenon. In combination with the annotation of genes adjacent to the DNA methylation sites, 637 gene promoter regions contained these HyperM sites. While in the neighborhood of the 1159 HypoM sites in the arsenic-exposure group, we isolated a total of 683 genes that met the criteria (Table 1).

**Table 1:** Analysis results of the aberrant DNA methylation sites

	CpG Site	Adjacent Genes
HyperM	1148	637
HypoM	1159	683
Total	2307	1279

This result indicates that the transcription status of these genes may be affected by arsenic treatment. It is noteworthy that in all of the annotation results, we found 41 genes with promoter regions that contained both HyperM sites and HypoM sites simultaneously.

### Pathway enrichment analysis of the genes adjacent to the aberrant DNA methylation sites

**Figure 1:**
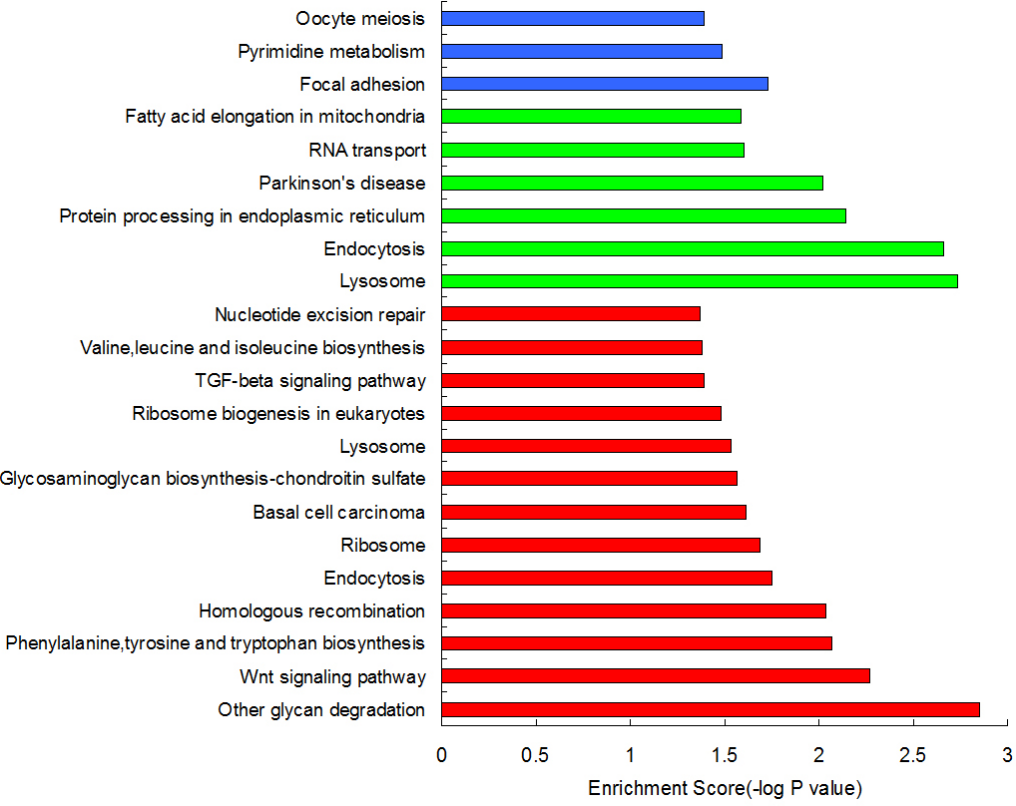
The pathway enrichment analysis results of aberrantly methylated genes in the promoter region The red columns represents HyperM adjacent genes, the green columns represent HypoM adjacent genes, and the blue ones represent Mixed genes.

Based on the above analysis results, we used the types of aberrant DNA methylation sites in the gene promoter region as a standard and categorized the genes adjacent to the aberrant DNA methylation sites as genes adjacent to hypermethylated sites (HyperM adjacent genes), genes adjacent to hypomethylated sites (HypoM adjacent genes) and mixed types of genes (Mixed genes, i.e., the promoter region contained both HyperM sites and HypoM sites at the same time). We performed a pathway enrichment analysis for each category. The detailed results for the pathway enrichment of each type of gene were shown in Figure 1.

Based on the KEGG pathway enrichment analysis results, we found that some HyperM adjacent genes in the arsenic-exposure group were enriched in the Wnt signaling pathway (including *MYC, APC, CACYBP, CAMK2G*, FZD7, *LEF1, LRP6, NFATC3, ROCK2, WNT2B* and *WNT7A*) and the TGF-beta signaling pathway (including *MYC, ACVR1C, FST, ROCK2, TGFBR2* and *THBS1*). In addition, genes related to the cellular metabolic pathways, such as phenylalanine, tyrosine and tryptophan biosynthesis, glycosaminoglycan biosynthesis, valine, leucine and isoleucine biosynthesis, may also be affected by arsenic-induced DNA HyperM. It is noteworthy that in the arsenic-exposure group, we found 4 nucleotide excision repair pathway genes, including *ERCC2, POLD3, POLE2* and *RPA1*. In addition, we found that HyperM adjacent genes were enriched in the ribosome pathway.

HypoM adjacent genes were enriched in the pathways of lysosome, endocytosis, protein processing in the endoplasmic reticulum, RNA transport, and fatty acid elongation in the mitochondria, whereas Mixed genes were mainly enriched in the pathways of focal adhesion, pyrimidine metabolism, oocyte meiosis, carbohydrate digestion and absorption.

In addition, in combination with the information from the Disease Ontology database, we found that 12 known primary hepatic cancer (PHC) related genes, including *MYC, CDC16, DDR1, EBAG9, IGF2, KDR, MAD2L2, MEF2C, NDRG2, SIX1, TNFRSF10B* and *TSGA10*, were HyperM in the arsenic-exposure group.

### Screening of transcription factors (TF) and tumor-associated genes (TAG) from genes adjacent to aberrant DNA methylation sites

In combination with known gene function information, we performed screening on HyperM adjacent genes, HypoM adjacent genes and Mixed genes. Additionally, we analyzed TF, oncogenes, tumor suppressors and other tumor-related genes without known functions from each type of gene. The details of the analysis results were shown in Table 2.

**Table 2:** Results of the functional screening of the genes adjacent to the aberrant DNA methylation sites

Class	TF	Oncogene	Tumor Suppressor	Other TAG
HyperM adjacent gene				
	*ARNTL2, BRCA2, ELF3, ERCC2, ETV7, FOXP1, GTF3A, HEYL, HNF4A, HOXA11, HOXC13, HOXD11, KCNIP3, LEF1, MEF2B, MEF2C, MYC, NFATC3, NPAS1, PBX1, SIX1, TAF10, TAF5, TBP, TBX22, TCEB1, TEAD4, TFAM, ZNF76*	*CEP55, COPS5, LF3, GNAS, MYC, PBX1, TAF15*	*APC, BECN1, BLCAP, BRCA2, CLU, COL18A1, CYLD, DLG1, EEF1A1, FOXP1, H19, L3MBTL4, LIMD1, MIR124–2, MIR17, MOB1A, NDRG2, PACRG, PTPN13, RND3, RPL10, SFN, TGFBR2, THBS1, TNFRSF10B, TRIM3, ZC3H10*	*DDR1, ERBB4, EVI2B, FZD7, NCOA4, WNT2B*
HypoM adjacent gene				
	*AHR, FOXD1, FOXI1, FOXJ1, FOXL2, HDAC5, HES1, HLTF, HMGA2, HOXA4, HOXC10, HOXC9, IRF7, LDB1, MEIS2, MXI1, NFE2L2, NKX2–1, RELB, RXRB, SMAD7, SMARCA4, SNAPC1, SOX14, STAT4, TEF, THRB, TMF1, TP73, ZNF143*	*CDK4, CTTN, ELL, FUS, HMGA2, NET1, NKX2–1, RALA, ZNF217*	*BARD1, BRMS1, DEFB1, FBXO31, HLTF, KLF6, MXI1, NDUFA13, PAF1, PHF6, PRR5, PTEN, PTENP1, RASSF5, SMARCA4, SUFU, TGFBR3, TP53BP2, TP73*	*ATP5B, BAMBI, BUB1, CTSZ, DHX16, DNMT3B, LAMP3, MBLAC1, NTN1, PCDH1, TFAP2A, TGFB1, THRB*
Mixed gene				
	*ETV6, SIX5, ZNF24*	*GFI1*	*ETV6*	

TF: transcription factors, TAG: tumor associated gene.

### Construction of the PPI (protein-protein interaction) sub-network of the genes adjacent to the aberrant DNA methylation sites

**Figure 2:**
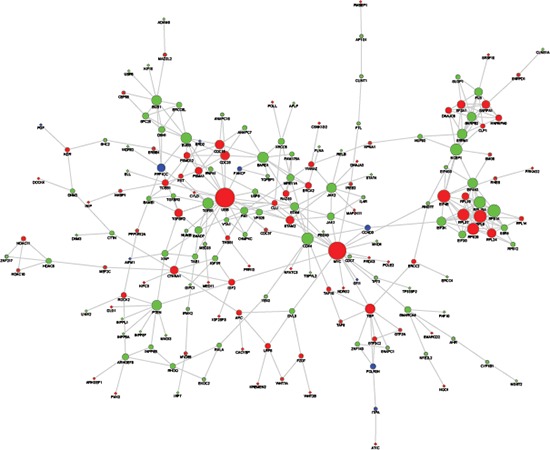
PPI sub-network of genes related to the aberrant DNA methylation sites The red nodes represent HyperM adjacent genes in the arsenic-exposure group, the green nodes represent HypoM adjacent gene, and the blue ones represent Mixed genes. The diameter of the node is positively proportional to the degree of that node.

Based on the String PPI database search results, we constructed a PPI sub-network of the genes adjacent to the aberrant DNA methylation sites (Figure 2). The results showed that in the relevant PPI sub-network, the key Hub genes with a node degree ≥ 10 included *UBB* (degree = 20), *MYC* (degree = 18), *EIF4E* (degree = 12), *RPL6* (degree = 12), *RPS14* (degree = 11), *NCBP1* (degree = 4), *RPL18A* (degree = 11), *NCBP1* (degree = 11), *RPL37* (degree = 11), *CDK4* (degree = 11 ), *RPS29* (degree = 11), *BARD1* (degree = 10), *BUB3* (degree = 10), and *TGFB1* (degree = 10).

### Annotation and enrichment analysis of the aberrant DNA methylation region related cis-regulatory sites

The detailed annotation results of the aberrant DNA methylation region related cis-regulatory sites are shown in Table 3. We demonstrated that one or more cis-regulatory sites existed in the neighboring regions of the 804 HyperM sites and the 834 HypoM sites. Considering that DNA methylation in the promoter region of the gene has the closest relationship to the transcriptional regulation of this gene, we performed further screening and collected all of the annotation results of the aberrant DNA methylation sites in the promoter region of the genes and their cis-regulatory elements. We used an enrichment analysis to detect the enrichment significance of each cis-regulatory element. The results showed that we identified 550 HyperM sites and 600 HypoM sites that met the abovementioned screening criteria.

**Table 3:** Compilation of annotation results of the cis-regulatory sites that are related to aberrant DNA methylation regions

	All CpG site covered one or more by cis-element	Related cis-element count	Promoter CpG Site covered one or more by cis-element	Related cis-element count
HyperM	804	71	550	66
HypoM	834	78	600	72

**Figure 3:**
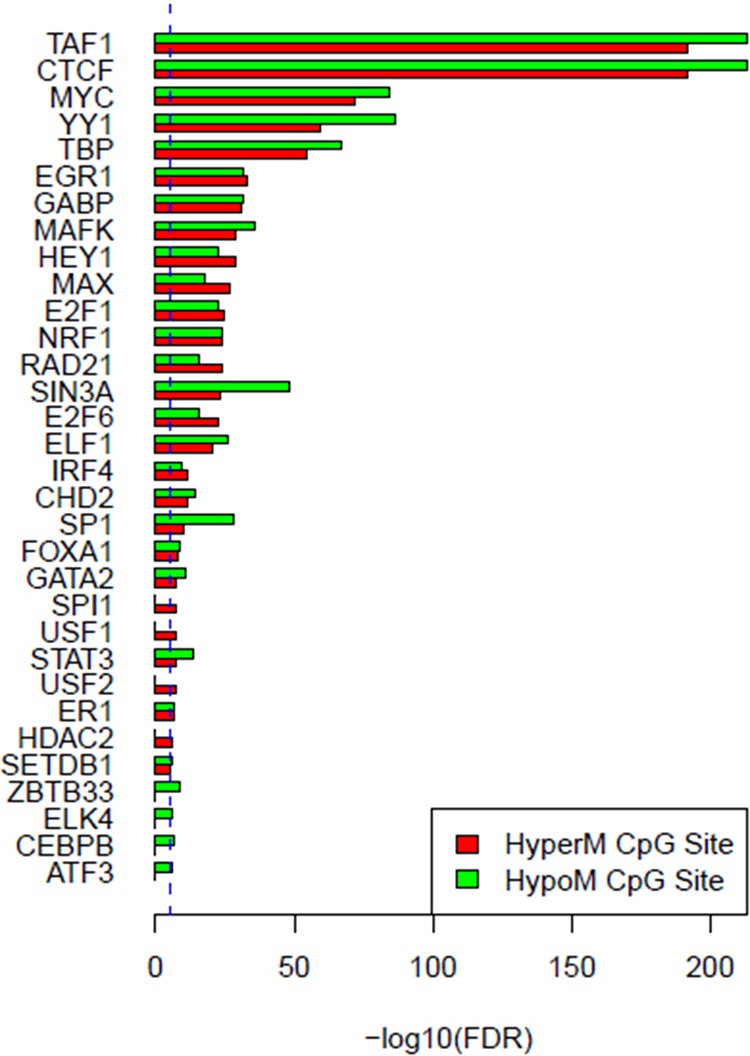
Enrichment analysis results of cis-regulatory elements of the aberrant methylation sites in the promoter region The red columns represent HyperM sites, and the green columns represent HypoM site. The blue dotted line is the enrichment significance threshold level, anything above this level indicates a significant enrichment result of the cis-regulatory element.

Based on the enrichment analysis of the annotation results of aberrant DNA methylation sites in the promoter region, we showed that the enrichment around the aberrant DNA methylation sites of a total of 32 TF was significant. Among these factors, there were significant enrichment phenomena around both HyperM and HypoM sites in the binding sites of 24 trans-acting factors, including *MYC, MAX, MAFK, CTCF, TAF1, IRF4, RAD21, E2F6, GABP, ELF1, SETDB1, HEY1, SIN3A, GATA2, ER1, TBP, STAT3, E2F1, CHD2, SP1, YY1, FOXA1, NRF1*, and *EGR1*. The cognate binding sites of 4 factors, *HDAC2, USF1, USF2* and *SPI1*, only had significant enrichment in the HyperM sites, whereas the cognate binding sites of 4 factors, *ATF3, CEBPB, ELK4* and *ZBTB33*, only had significant enrichment in the HypoM sites in the arsenic-exposure group (see Figure 3). The results of the classification of these 32 trans-acting factors according to their functions showed that they included 21 TF, 3 known oncogenes, 4 known tumor suppressor genes and 3 TAG. The details of each type of gene are listed in Table 4. Meanwhile, the results of the analysis based on the TF motifs around the aberrant DNA methylation sites showed that the DNA binding sites of a total of 6 TF, including *MYC, MAX, USF1, TAF1, USF2* and *E2F1*, were enriched in the surrounding region of these DNA methylation sites (see Table 5). This result is consistent with the enrichment analysis results of the cis-regulatory sites.

**Table 4:** Functional classification of trans-acting factors corresponding to significantly enriched cis-regulatory elements

	Gene Count	Genes
TF	21	*ATF3, ELK4, USF2, MAFK, CTCF, TAF1, IRF4, HDAC2, MAX, ELF1, HEY1, GATA2, TBP, SPI1, USF1, STAT3, SP1, YY1, FOXA1, NRF1, EGR1*
Oncogene	3	*MYC, IRF4, SPI1*
Tumor Suppressor	4	*CTCF, SIN3A, E2F1, EGR1*
Other TAG	3	*MAX, SETDB1, STAT3*

TF: transcription factors, TAG: tumor associated gene.

**Table 5:** Results of the enrichment analysis of transcription factor motifs in the neighborhood of aberrant DNA methylation sites

TF	Motif	MDSeqpos enrichment score (−log *P* value)
*MYC*	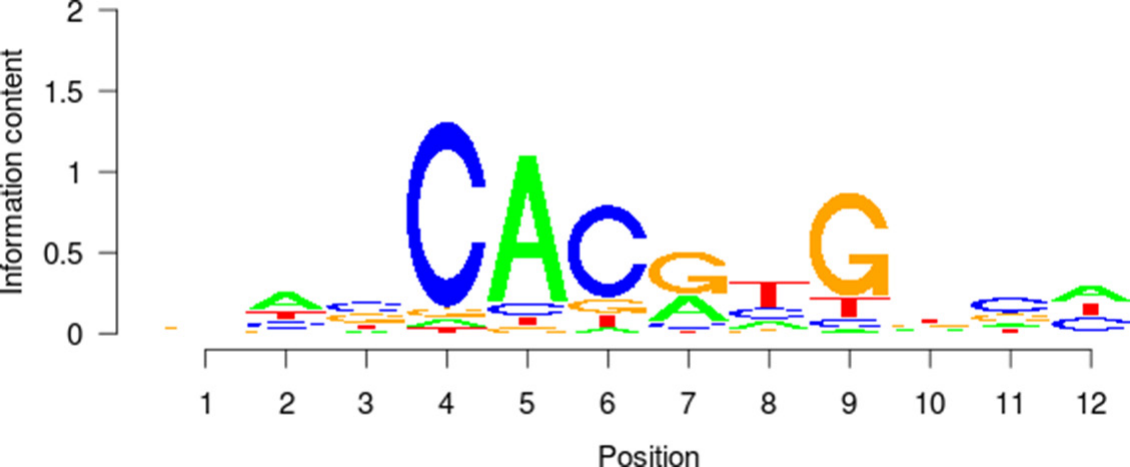	142.211
*MAX*	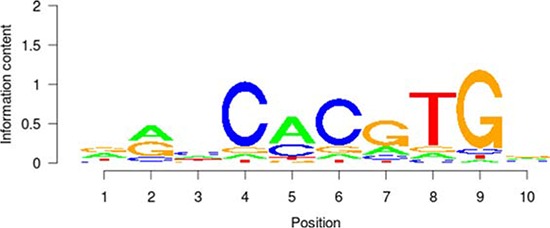	124.265
*USF1*	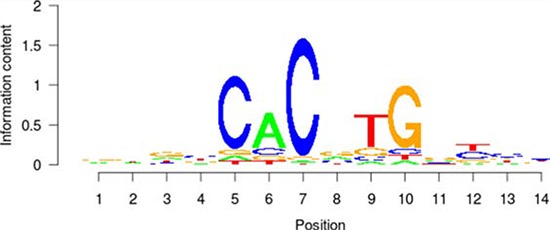	122.075
*TAF1*	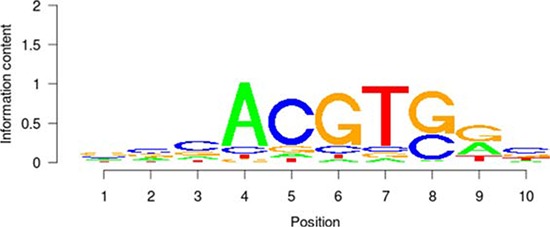	171.884
*USF2*	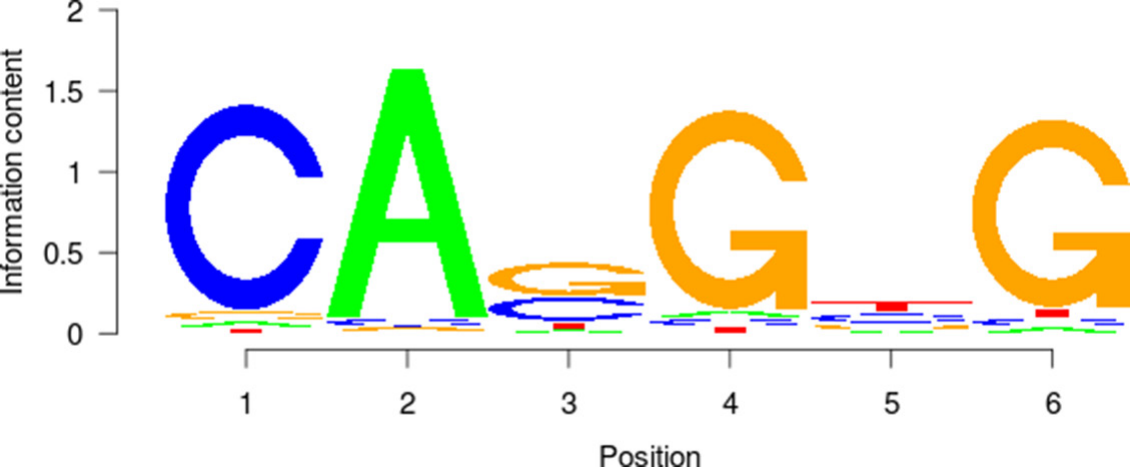	130.845
*E2F1*	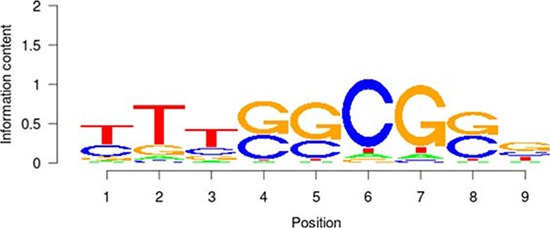	122.043

TF: transcription factors.

### Construction of aberrant DNA methylation site related transcriptional regulation network

**Figure 4:**
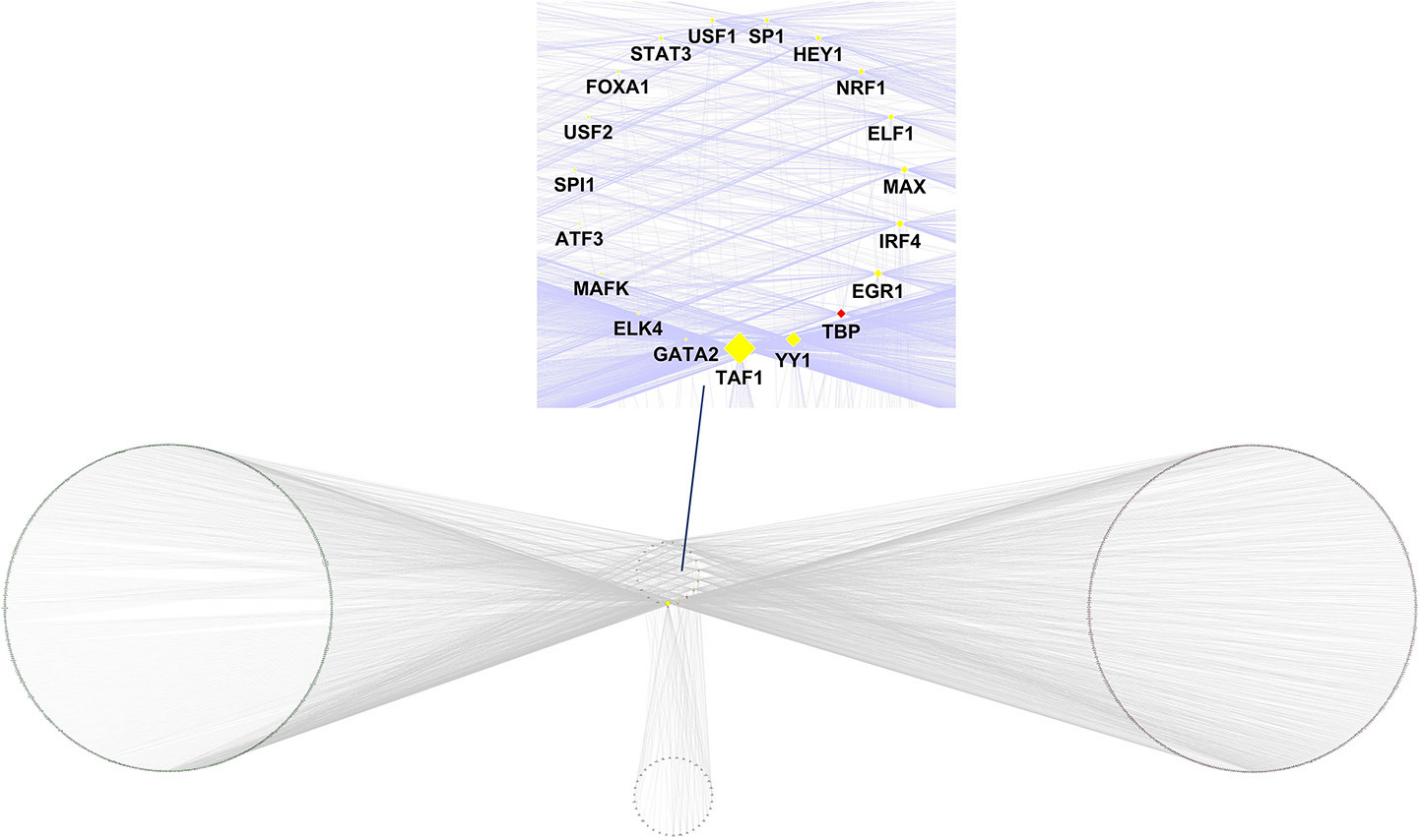
Transcriptional regulatory network that is related to the aberrant DNA methylation sites

Based on the annotation results of the genes adjacent to the aberrant DNA methylation sites and the enrichment information of TF binding sites from the enrichment results of the cis-regulatory elements of the aberrant DNA methylation sites, we further constructed an aberrant DNA methylation site related transcriptional regulation network (Figure 4). This transcriptional regulation network contained a total of 2290 TF downstream target gene pairs, of which the cis-regulatory element enrichment results included 21 TF, corresponding to 961 target genes. In this network, the network core genes with the 10 highest node degrees in order were *TAF1* (degree = 803), *YY1* (degree = 338), *TBP* (degree = 184), *EGR1* (degree = 159), *IRF4* (degree = 148), *MAX* (degree = 126), *ELF1* (degree = 96), *NRF1* (degree = 87), *HEY1* (degree = 83), and *SP1* (degree = 64).

### Detection results of *MYC* and *MAX* mRNA expression

**Figure 5:**
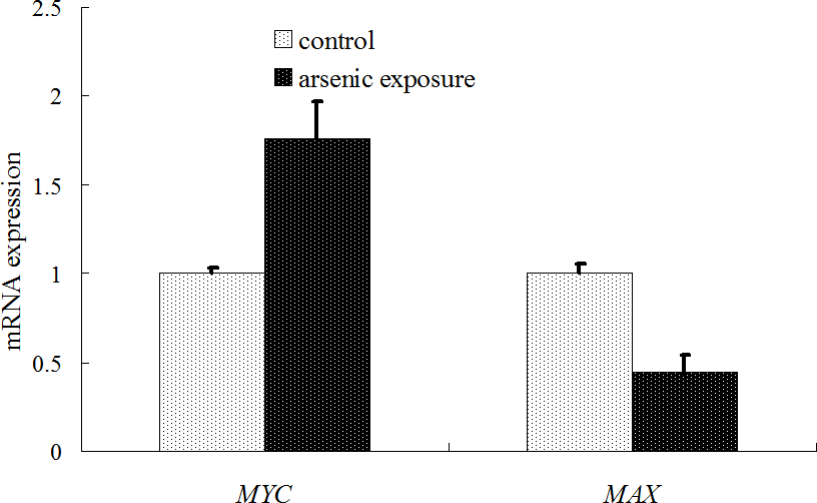
Diagram of the detection results of *MYC* and *MAX* mRNA expression using real-time PT-PCR

The mRNA expression of *MYC* and *MAX* in the liver cells in the arsenic exposure group and the control group was examined using a real-time RT-PCR method. The results were shown in Figure 5. *MYC* mRNA expression was significantly increased in the arsenic-exposure group (1.76 ± 0.21 *vs*. 1.0 ± 0.03, *P* < 0.05), whereas *MAX* mRNA expression was significantly decreased in the arsenic-exposure group (0.45 ± 0.09 *vs*. 1.0 ± 0.05, *P* < 0.05).

## DISCUSSION

In the present study, we found that arsenic treated liver cells had significantly increased aberrant DNA methylation. DNA methylation is a typical epigenetic marker. Under the catalysis of methyltransferase, the cytosine of the CG dinucleotides in DNA is selectively modified by a methyl group, forming 5-methyl-cytosine. DNA methylation can induce changes in DNA structure and stability and can affect gene transcription and expression. Many studies have shown that DNA methylation can promote or inhibit the pathogenesis of tumors [8]. A study by Nishida N [9] reported that methylation in the promoter region of a gene may inactivate the corresponding tumor suppressor gene and that the genome instability likely caused by the HypoM of the entire gene also activates the pathogenesis of cancer. Omura K [10] noted that the methylation and expression of normal liver genes and the immune response are involved in early carcinogenesis. Therefore, aberrant DNA methylation is an important mechanism for the pathogenesis of cancer.

Inorganic arsenic can interfere with DNA methylation in its *in vivo* metabolic processes [11]. As an enzyme inhibitor, inorganic arsenic selectively inhibits S-adenosylmethionine-dependent methyltransferase, thereby reducing the utilization of S-adenosylmethionine and causing its concentration to increase, whereas the uninhibited methyltransferase HyperM cytosine [12]. Likewise, some reports have shown that inorganic arsenic can also lead to the HypoM of genes. Its mechanism could be that inorganic arsenic consumes the methyl group of S-adenosylmethionine, causing insufficient intracellular methyl group status, thereby leading to the instability of the methylation mode and the demethylation of genes [13]. Additionally, HyperM and HypoM gene can co-exist to regulate gene functions.

In the present study, through the KEGG pathway enrichment analysis of genes adjacent to aberrant methylation sites, we found that the DNA methylation levels in the promoter regions of 4 nucleotide excision repair pathway genes (*ERCC2, POLD3, POLE2* and *RPA1*) were higher than in the control group. The DNA repair ability binds to maintaining the human genome integrity and stability and cellular functions. Some reports noted that when DNA repair related genes were inhibited, the sensitivity to cancer causing factors would increase [14]. Studies by Abbasi R [15] showed that the down-regulation of nucleotide excision repair genes would significantly increase the risk of laryngeal cancer. Long-term exposure to arsenic may lead to the transcriptional inhibition of genes related to chromosome base repair inside the cell, making the cells fail to maintain their ability to correct and repair base errors. For example, some studies reported that the *ERCC2* promoter was HyperM in arsenic treated hepatocytes, which inhibited the CAK (cyclin-dependent protein kinase activating kinase)-complex and eventually suppressed DNA repair. This could be one of the important factors for long-term arsenic exposure induced liver cancer [16].

**Figure 6:**
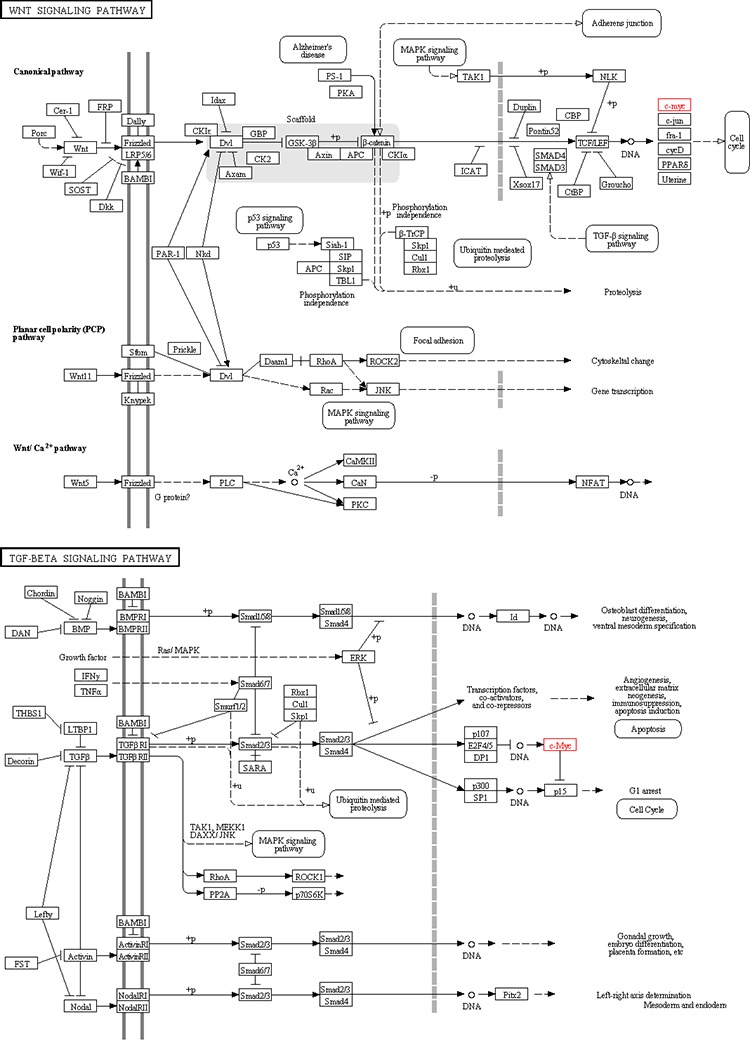
Map of Wnt signaling pathway and TGF-beta signaling pathway

In the present study, we found that HyperM adjacent genes in the arsenic exposure group were mainly enriched in the Wnt signaling pathway and the TGF-beta signaling pathway. Reports showed that these signaling transduction pathways participating the pathogenesis and progression of liver cancer [17, 18]. As shown in Figure 6, the Wnt signaling pathway and the TGF-beta signaling pathway pass extracellular molecular signals through the cell membrane to the inside of the cell to exert their effects. Both pathways are involved in activating cell cycle, and *MYC* is located downstream of both pathways, which is a key factor in regulating the cell cycle. Therefore, *MYC* should be a key node of the present study.

Tumorigenesis is inextricably linked to the changes of oncogenes, particularly proto-oncogenes and tumor suppressor genes. Many studies have confirmed that the activation and excessive expression of proto-oncogenes can promote tumor formation. Meanwhile, the loss or inactivation of tumor suppressor genes may also lead to tumorigenesis. Therefore, we screened TF, proto-oncogenes, tumor suppressor genes, and other TAG from the genes adjacent to aberrant DNA methylation sites, of which multiple known cancer related genes, including *MYC, DDR1, MEF2C, NDRG2, SIX1, TNFRSF10B* and *TSGA10*, had HyperM phenomena. In addition, from the PPI sub-network of the genes adjacent to the aberrant DNA methylation sites, we found that among the key genes with a gene node degree greater or equal to 10, *MYC* [19], *EIF4E* [20], *CDK4* [21] and *TGFB1* [22] could stimulate the pathogenesis of liver cancer.

However, according to the functional analysis of the abovementioned genes adjacent to the aberrant methylation sites, we found that the adjacent site of the proto-oncogene *MYC* was in a HyperM state. Based on this observation, we inferred that the transcription of *MYC* should be inhibited and *MYC* mRNA expression should be reduced. However, as a proto-oncogene, *MYC* should be increased in its carcinogenic process [23]. Jang KY [19] reported that the high expression of *MYC* could promote the pathogenesis of liver cancer and that *MYC* was considered an indicator of the prognosis of liver cancer. Our real-time RT-PCR detection results also showed that the expression of *MYC* mRNA increased after long-term exposure to arsenic (Figure 5). Obviously, a simple functional analysis of genes adjacent to aberrant methylation sites cannot well explain the effects of DNA methylation on gene transcription during arsenic induced carcinogenic processes.

Therefore, we further performed the annotation and enrichment analysis of cis-regulatory sites that are related to aberrant DNA methylation regions. DNA methylation can result in conformational changes of DNA in the same region on the chromosome, thereby affecting the interaction between the protein and DNA and inhibiting the binding efficiency of the trans-acting factor to DNA. Therefore, we combined the information of the binding sites on the chromosome of all known trans-acting factors, and we searched and analyzed the cis-regulatory sites (i.e., binding sites) of the known trans-acting factors, including TF binding proteins, enhancer binding proteins and insulator proteins, in the proximal regions of both HyperM sites and HypoM sites. We combined enrichment analysis methods to further screen the regulatory elements that were significantly affected by aberrant DNA methylation in the arsenic exposure group.

It is noteworthy that the functional classification of trans-acting factors, whose corresponding cis-regulatory elements are significantly enriched, showed that *MYC* is an important oncogene (Table 4). The results of the enrichment analysis of the TF motifs surrounding the aberrant DNA methylation sites indicated that the MDSeqpos enrichment score of *MYC* was 142.211 (the default enrichment significance threshold was 0.00001, see Table 5). In combination with the known information recorded in the Disease Ontology database, we found in Figure 3 that the cis-regulatory element of *MYC* had both HyperM sites and HypoM sites simultaneously, and the HypoM was predominant. Based on this result, we can infer that the expression of *MYC* should be up-regulated. This result is exactly consistent with our RT-PCR detection results (Figure 5) and literature reports.

Recent studies showed that MYC gene family proteins and their associated proteins MAX (MYC-associated factor X) form a network to regulate cell cycle. When these regulatory factors are abnormal, it will lead to uncontrolled cell proliferation and differentiation and the formation of tumors [24, 25]. Ecevit O [26] and Adhikary S [27] reported that known MYC functions required the formation of a dimer with MAX to work. MYC can form a stable heterodimer with MAX and activate the transcription of the correlated gene. During the entire cell cycle, with the change of MYC and MAX protein levels, either MYC-MAX activates transcription or MAX-MAX inhibits transcription and replication [28]. Studies have shown that many malignant tumors have over-expressed MYC and under-expressed MAX [29]. Cascon A [30] reported that the lack of MAX in PC12 cells (derived from rat adrenal pheochromocytoma) make it easier to experience tumorigenesis, Albanus RD [31] reported that the expression level of MAX is positively correlated to the survival rate of breast cancer and lung cancer patients, Comino-Méndez I [32] reported that the loss of function of MAX would increase the invasiveness of pheochromocytoma, Yang L [33] reported that the loss of expression of MAX may activates the pathogenesis of pancreatic cancer.

In the present study, the functional classification of trans-acting factors, whose corresponding cis-regulatory elements are significantly enriched, showed that both TF and TAG contain *MAX* (Table 4). The results of the enrichment analysis of the TF motifs surrounding the aberrant DNA methylation sites indicated that the MDSeqpos enrichment score of *MAX* was 124.265 (the default enrichment significance threshold was 0.00001, see Table 5). After the construction of the transcriptional regulation network, which was related to the aberrant DNA methylation sites, we found that *MAX* (degree = 126) was one of the most important network core genes (Figure 4). In the analysis in Figure 3, the *MAX* cis-regulatory element contains both HyperM sites and HypoM sites, and the HyperM was dominant. We speculated that *MAX* transcription would be inhibited and *MAX* mRNA would be expressed at a low level. Our real-time RT-PCR also detected the low expression of *MAX* mRNA (Figure 5). This indicates that during the long-term arsenic exposure process, the HypoM of *MYC* up-regulates *MYC* expression and the HyperM of *MAX* down-regulates *MAX* expression, thereby increasing the carcinogenesis tendency of hepatocytes.

In this study, based on the DNA methylation microarray analysis results under long-term arsenic exposure conditions and the results of the database search and literature search, we found that some genes adjacent to DNA methylation were related to the pathogenesis of liver cancer. The analysis of cis-regulatory elements and their trans-acting factors that are adjacent to DNA methylation sites can more accurately reflect the correlation between methylation and gene transcription and expression. We deduced that the HypoM of *MYC* and the HyperM of *MAX* may activate the pathogenesis of arsenic induced liver cancer.

## MATERIALS AND METHODS

### Cell culture

The normal human liver cell line L02 was purchased from the China Center for Type Culture Collection (CCTCC, Wuhan, China). The cells were cultured in DMEM (high glucose) supplemented with 10% FBS at 37°C and 5% CO_2_ in an incubator.

The medium for the arsenic exposure group was supplemented with a low concentration of arsenic trioxide, which was purchased from Yida Pharmaceutical Co. Ltd., Harbin Medical University, Harbin, China. The final concentration was 0.2 μM. The cells were split every 48 h, and a fresh arsenic-containing medium was used. After 3 months of culture, the cells were harvested for detection. The control group cells were cultured in DMEM medium only, and the cells were harvested for detection at the same time point. All experiments were performed in triplicate.

Approval for this study was obtained from the Committee for Medical Research Ethics of the First Affiliated Hospital of Harbin Medical University, which are the authority of research ethics in China.

### Infinium® HumanMethylation450 BeadChip microarray

**Figure 7:**
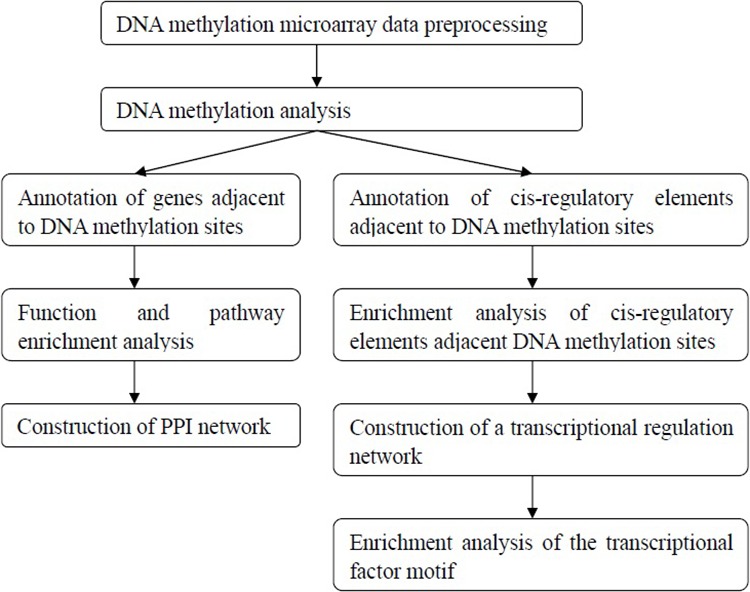
The bioinformatics analysis flowchart of the DNA methylation microarray detection results

DNA was isolated using a DNeasy Blood & Tissue Kit (Qiagen, Hilden, Germany, Cat No. 69504), the DNA samples were quantified, and their quality was examined. The DNA samples from each group were mixed together for further detection. An Infinium® HumanMethylation450 BeadChip (Illumina Inc, San Diego, CA, USA) was used for the transformation, amplification and labeling hybridization experiments. The procedures were conducted according to the Illumina Infinium® HumanMethylation450 BeadChip operating manual. Then, bioinformatics analyses were performed followed the flowchart as shown in Figure 7.

### DNA methylation microarray data preprocessing

The Methylumi package [34] was used to read the raw microarray data in .idat format. The Lumi package [34] was used to complete the pretreatment operation for the Illumina Infinium DNA methylation microarray, which included dual-channel microarray fluorescence signal value imbalance adjustment (color imbalance adjustment, quantile based), background signal correction (background correction), and data normalization (quantile based). Afterwards, the beta value of each DNA methylation site was calculated to reflect the DNA methylation level at each probe site. The formula for the calculation of the beta value was as follows:

$$Be\,t\,a_{i}=\frac{\text{max}(\;y_{i,m,e,t,h,y},0)}{\text{max}(\;y_{i,u,n,m,e,t,h,y},0)+\text{max}(\;y_{i,m,e,t,h,y},0)+\alpha }$$

In which *i* represents the serial number of each probe on the chip, and α is the compensation value. Here, the default value of α was set to 0.

### Detection of aberrant DNA methylation sites

Based on the pretreatment results of the microarray, the differences of the beta value of each DNA methylation site in the arsenic exposure group and in the control group were compared one by one. We ensured that the methylation sites, whose fold coefficient differences of DNA methylation levels between samples were not smaller than 1.5 (i.e., | log2 (arsenic exposure group vs. control group) | ≥ 0.58), were significantly aberrant sites.

### Annotation of genes adjacent to the aberrant DNA methylation sites

The region between 2 kb upstream of TSS (transcription start site) and 0.5 kb downstream of TSS of each gene was defined as the promoter region of the gene. Thus, combined with the coordinate information of the probe, we determined whether each probe was within the promoter region of the gene. Aberrant DNA methylation sites that met the criteria were kept, and the relevant gene was defined as the adjacent gene of the aberrant DNA methylation sites.

### Pathway enrichment analysis of the genes adjacent to the aberrant DNA methylation sites

The enrichment of pathways of the genes adjacent to the aberrant DNA methylation sites were identified based on the information from the KEGG pathway database [35]. During the analysis, the default significant threshold value of the hypergeometric test enrichment was set at 0.05, and the number of genes included in each significantly enriched KEGG pathway was not less than 2.

### Screening of genes with specific functions

In combination with the TF data information recorded by TRANSFAC [36], further screening and annotation were performed on the genes adjacent to the aberrant DNA methylation sites to determine whether these genes had transcriptional regulation functions. Second, in combination with the tumor suppressor genes [37] and the TAG database [38], we further screened for all known oncogenes and tumor suppressor genes.

### Construction of a sub-network of genes adjacent to aberrant DNA methylation sites based on PPI network

Based on the information provided by the String database [39], we collected other protein-encoding genes that can interact with these genes and established a PPI sub-network. Only those interacting protein pairs that had been verified by experiments, had been reported before, had previously undergone a co-expression analysis or had been recorded in a relevant database were selected as the input data for the sub-network construction. The PPI value of each interacting protein pair was not lower than 0.9 (PPI score was between 0 and 1; the PPI value was positively correlated with the PPI interaction pair credibility).

### Annotation and enrichment analysis of cis-regulatory elements adjacent to aberrant DNA methylation sites and the construction of a transcriptional regulation network

First, based on the ENCODE database [40], information regarding the cognate cis-regulatory binding sites of all known human trans-acting factors was extracted. Second, to enhance the credibility of these cis-regulatory binding sites, we statistically analyzed the reproducibility of each binding site, and the cis-binding sites that showed up in at least two independent samples were selected and used for subsequent analysis. Then, in combination with relevant DNA methylation site data, we selected all the cis-regulatory regions that overlapped with DNA methylation sites and defined these regions as the cis-regulatory elements adjacent to DNA methylation sites.

Based on the results from the above treatment, we annotated the known cis-regulatory sites that were located around the aberrant DNA methylation sites. In combination with Fisher's exact test (FET) enrichment analysis, we performed further screening to predict the transcriptional regulatory elements that were greatly affected by DNA methylation. Based on this information, we built a transcriptional regulatory network. The FET enrichment significance threshold was set to 0.00001.

### Enrichment analysis of the TF motif of the aberrant DNA methylation sites

First, the aberrant DNA methylation site was used as the center and the region consisting of 50 bp upstream and downstream of the site was used as the detection range for the motif analysis. Then, in combination with each TF DNA binding motif properties that were recorded in the TRANSFAC [36] and JASPAR databases [41], we used the MDSeqpos tool to perform a TF motif enrichment analysis in the detection region. The default enrichment significance threshold was set at 0.00001.

### Detection of *MYC, MAX* mRNA using real-time RT-PCR

Total RNA was extracted using the RNeasy Kit (Qiagen, Hilden, Germany) according to the manufacturer's instructions. RNA quantity and quality were measured by NanoDrop ND-1000 and RNA integrity was assessed by standard denaturing agarose gel electrophoresis. Freshly prepared RNA (1mg) was reverse transcribed using the HiFi-MMLV cDNA reverse transcription kit (CWbiotech, Beijing, China, Cat No. CW0744) and RealSuper Mixture (with Rox) (CWbiotech, Beijing, China, Cat No. CW0767) according to the manufacturer's instructions. All reactions were performed on an ABI Prism 7500 Real-Time PCR system (Applied Biosystems, Foster City, USA). *MYC*, primers: forward (TCAAGAGGTGCCACGTCTCC), reverse (TCTTGGCAGCAGGATAGTCCTT), and probe (CAGCACAACTACGCAGCGCCTCC). *MAX*, primers: forward (AGGTGGAGAGCGACGAAGAG), reverse (GTGCATTATGATGAGCCCGTTT), and probe (CCGAGGTTTCAATCTGCGGCTGAC). Relative gene expression was normalized to 1.0 of controls. All experiments were performed in triplicate.
